# Artificial intelligence-driven automation is how we achieve the next level of efficiency in meat processing

**DOI:** 10.1093/af/vfac017

**Published:** 2022-04-30

**Authors:** Chafik Barbar, Phillip D Bass, Rachel Barbar, Jordyn Bader, Britany Wondercheck

**Affiliations:** 1 Marble Technologies, Cambridge, MA 02139, USA; 2 Department of Animal, Veterinary and Food Sciences, University of Idaho, Moscow, ID 83844, USA

**Keywords:** artificial intelligence, automation, meat processing, robotics

**Implications**

- The long-standing challenges to automation of meat processing, particularly the variability of meat products, are being solved by advancements in artificial intelligence technology, rather than advanced mechanical engineering.
- The next generation of automation will be a software product rather than a hardware product.
- Artificial intelligence-driven automation should be assessed through a strategic lens that looks beyond reduced labor costs.

## Introduction

Meat processors have long worked to optimize their operations. Historically a cyclical business characterized by lean margins, meat processors find it necessary to keep costs low and optimize profit per animal harvested to remain viable.

Optimization in meat processing has been achieved through a combination of ongoing initiatives. Worker specialization (Crespi and Saitone, 2019), by limiting the number of different tasks each person is responsible for, has made meat plant operations efficient and fast. Engineering of facility layouts and management of maintenance schedules have increased uptime and productivity. New technologies, like beef carcass grading camera imaging systems (Moore et al., 2010; Gray et al., 2012; Emerson et al., 2013), have been adopted to improve carcass value assignment and utilization. Warehouse inventory management systems, branding for premium products, and customer relationship management software all help meat packing facilities improve profitability.

Automation has also played a role in the optimization of meat processing. Poultry evisceration and deboning as well as chemical stunning of poultry and hogs have been successfully automated in commercial settings (Barbut, 2014). Beef and pork carcass automated splitting equipment has been developed for commercial use as well as automated carcass chilling. Even cut inventory management has reaped the benefits of automation (Barbut, 2020). Furthermore, commercially available modes of pork and lamb fabrication equipment are already accessible in the meat processing market (Frontmatec 2021a, 2021b); proof that automation has already made an indelible mark on the meat industry.

Additional optimization through automation via robotics, however, has been limited due to technical challenges. Traditional robotics excels when the task (and all its inputs) are consistent and predictable; for example, picking and placing parts of known size and shape. Animal carcasses and meat subprimals are not so consistent. Both shape and size vary, particularly in larger animals like beef (Boykin et al., 2017; Tarakji, 2018) making the application of robotic automation very challenging (Choi et al., 2013). Greater progress has been seen in automating fabrication of smaller and more uniform species, such as poultry or lamb, and in secondary processing, such as cutting chicken nuggets, as the variation the technology must overcome is decreased (Barbut, 2014; Barbut, 2020).

Carcass variation isn’t the only technical hurdle slowing the automation of meat animal carcass fabrication. Automated systems must keep pace with rapid line speeds and maintain high yields, all while ensuring food safety and occupational safety. Moreover, space within meat processing plants often is at a premium. Facility expansion or long downtimes for installation increase the cost of implementing new technology and slow down adoption.

In addition to technical challenges, other factors, such as labor costs and workforce shortages, also impact the cost-benefit analysis for automation of meat processing. Not surprisingly, regions of the globe with higher labor costs, particularly Australia and Europe, have made greater investments and seen more progress in meat processing automation. In the United States, escalating labor shortages and the COVID-19 pandemic have increased meat processors’ interest in automation (Byington, 2020). Companies around the world are now stepping up and leading efforts to develop and implement automation. They are ready to usher in the next level of optimization to meat processing.

## Next Phase of Automation: Artificial Intelligence-Driven Automation

### Perception, decision-making, and action

Every robot within an autonomously automated system has to do three things: perceive its environment, make decisions based on those perceptions, and act on its environment based on those decisions (Zahariev and Valchkova, 2019). During previous phases of automation, engineering effort focused on actuation and building the hardware to perform tasks where perception and decision-making are relatively easy. An example where automation has succeeded with limited perception and decision-making is in machine-tending where a robot picks a raw product part from a known location, fixtures it in a computer numerical controlled machine, and retrieves the completed part when the operation is finished. The exact size and shape of the part is known at all steps, simplifying the perception aspect, and the sequence of moves is identical on every repetition, reducing the decision-making load.

The newest phase of automation works to automate tasks where the challenge is not as much actuation but perception and decision-making. Perception and decision-making is accomplished by the automation’s software using artificial intelligence. Artificial intelligence is, “the art of creating machines that perform functions that require intelligence when performed by people (Kurzweil, 1990).”

In the context of meat processing, artificial intelligence allows the automated system to be responsive to the variation between different carcasses and subprimals. Using AI-driven software, an automated system can not only perceive differences, but also use those perceptions to make decisions about how to act. The use of AI allows for the automation of tasks that require intelligent decision-making, such as identifying and sorting subprimals or deciding where to trim surface fat on a particular cut of meat.

This is not to say that hardware doesn’t matter. Robotic arms, end effectors, and other pieces of equipment remain critical components of automation. The increasing availability of high-quality robots at competitive prices as well as advancements in this equipment, such as the decrease in robot size relative to its strength, are making automated systems technically and economically more feasible for the meat industry (Tedrake, 2021). Sensors, cameras, and other hardware components that are ruggedized to withstand the cold, wet, sanitized environment of the plant are also critical for AI-driven automation systems. Yet, the major new capabilities of automated systems will be the result of advancing the software “brains” to improve the system’s decision-making and actions.

### A new level of optimization

First evaluation of AI-driven automation seemingly focuses on a reduction in the number of workers required by a facility (Jackson et al., 2020). While this is one benefit, particularly for facilities facing significant labor shortages, it’s just the beginning.

#### Big data for meat processing.

So far, big data use is a missed opportunity for many meat processing scenarios. Massive amounts of data are generated each day in high-volume facilities, but it is rarely captured and aggregated in a meaningful way for real-time decision-making. An AI-driven automated system collects data for the perception and decision-making components of its task. Once collected, this data can also be analyzed for application to the optimization of other areas of the operation or to suggest process improvements that further maximize the processing capacity of existing assets.

**Figure 1.**
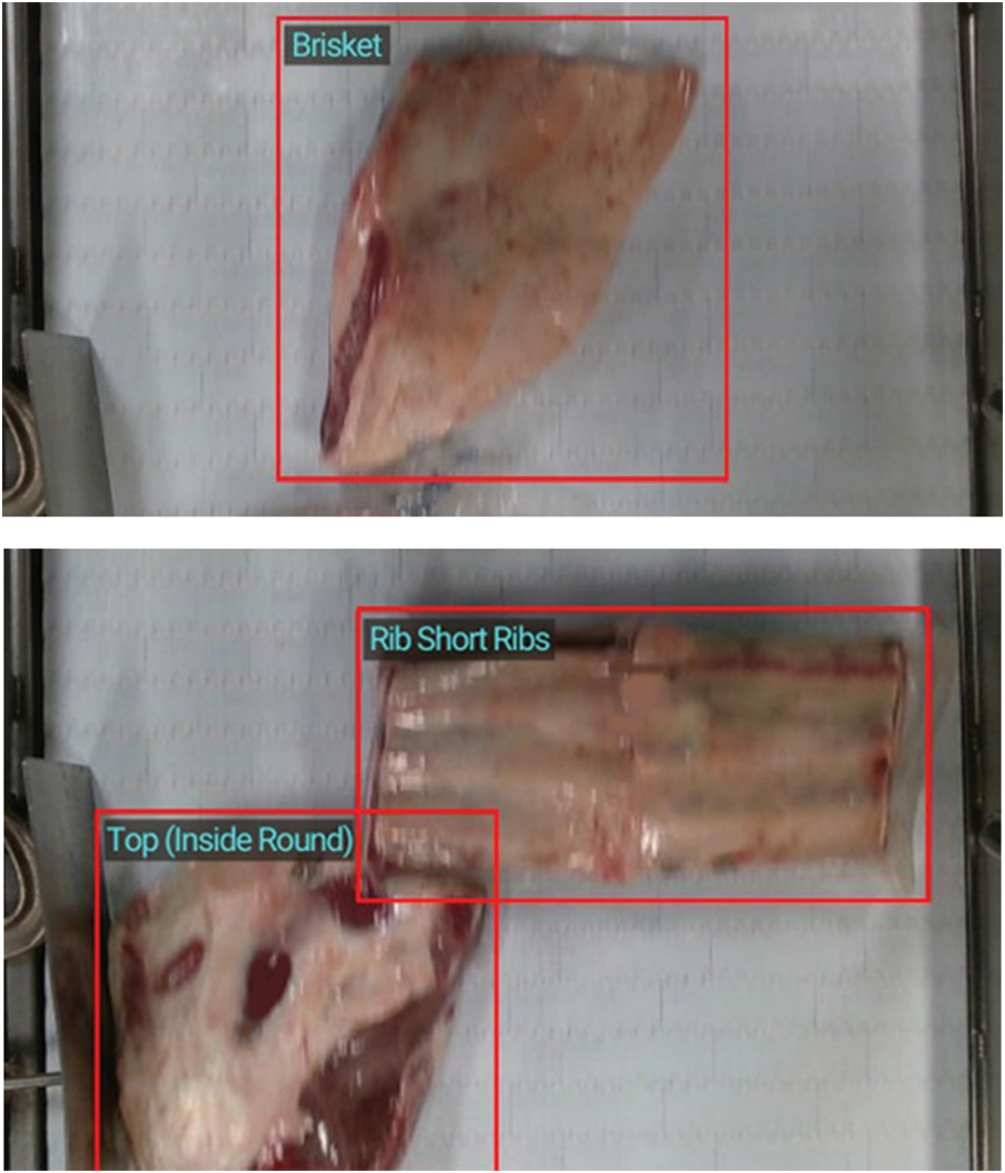
Automated computer vision technology identifying product for final boxing.

For example, AI-driven automated systems in meat processing might collect data about the amount of fat covering a subprimal, the weight and dimensions of a subprimal, whether or not there is contamination, or the identity of a specific product packaged into boxes (Figure 1). The real-time weight and dimensions of a subprimal might first be captured to help direct it to the correct workstation, but this real-time data might then also be applied to decisions directing the trimming and fabrication of the subprimal to reduce the number of lost yield opportunities and optimize the use of variable subprimals. Eventually, this data on size and dimensions could be tied back to the live animal production practices (e.g., genetics, nutrition, growth technologies, etc.) leading to further optimization of livestock production programs.

Big data can create a real-time, high-level viewpoint. Systems can begin to identify patterns in the data, including patterns humans might miss or only sometimes pick up (LeCun et al., 2015). With this vantage point, management has more robust and exact data for decision-making, helping them drive their operations to the next level of optimization. This path to greater optimization is made more exciting by its seemingly low cost. Once an automated system is installed and the data has been collected, the cost to develop and apply analytical tools is minimal.

#### Quality assurance.

Meat processors who implement technology driven by AI will also achieve greater consistency in product quality. AI-driven automation systems are being designed to produce products exactly to specification every time. These systems might also inspect package integrity, identify products that need to be reworked to meet specifications, or allow for verification that the correct product was shipped to the target customer. Such systems will not suffer from fatigue-induced errors or mistakes (Salonen and Haavisto, 2019), providing an opportunity to advance quality assurance and improve profitability.

#### Worker safety.

**Figure 2.**
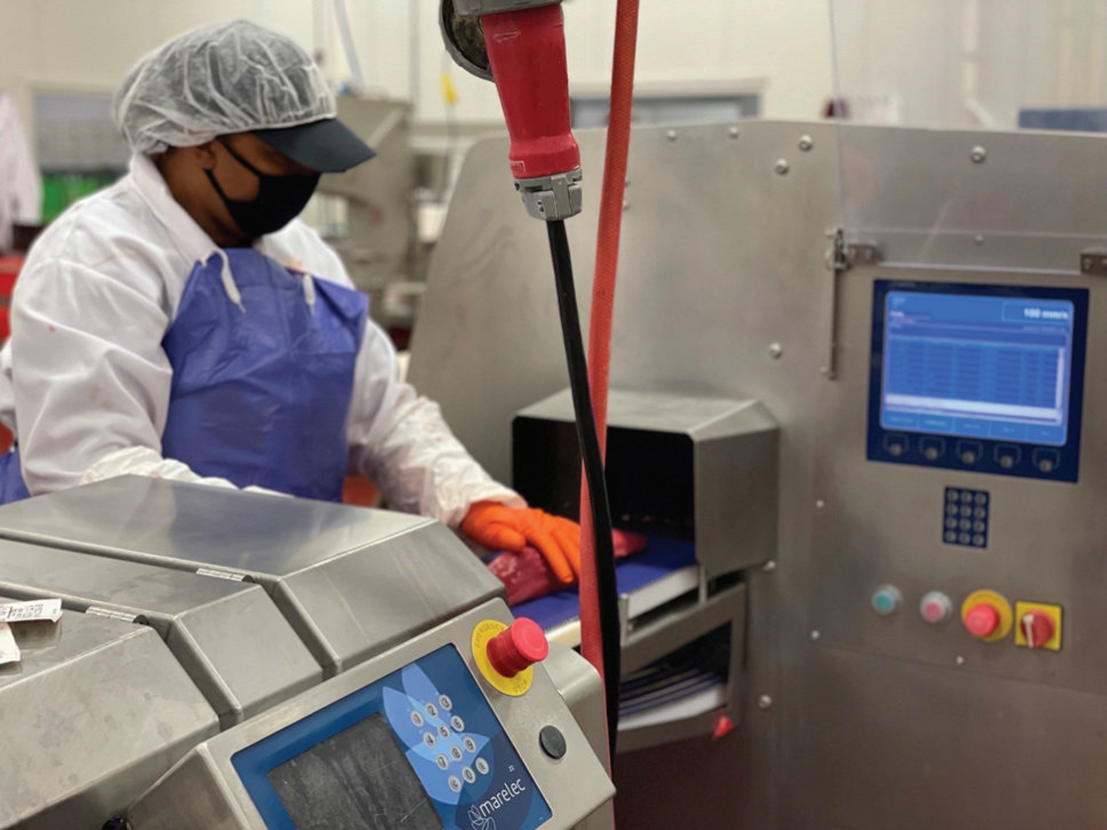
Process operator supervising an automated production system. (Image Credit: James Galione).

Artificial intelligence-driven automation increases worker safety. Improvements in worker safety are not simply a result of fewer workers in the facility. Rather, automation can be used to augment the more strenuous, repetitive, and dangerous tasks of human workers (Figure 2). As workers transition to roles supervising the technology, the likelihood of cuts, punctures, and repetitive motion injuries should decrease. The big data collected by artificial intelligence-driven automation may also generate insights that further improve worker safety (Howard, 2019) which may enhance both employee satisfaction and worker retention.

#### Fabrication redesigned.

AI-driven automation allows for the rethinking of the meat animal carcass fabrication process. Tools and equipment are not limited by human dexterity, concerns of bodily injury, or strength limitations. Different cutting tools, changed orientations, cuts made on the away side of the subprimal, even multiple cuts made in unison, are all possibilities. Unlike traditional automated systems, AI-driven automation introduces the possibility of a single robotic tool performing multiple tasks.

## Why AI-driven Automation Succeeds

AI-driven automation has advantages that extend beyond the ability to overcome technical challenges. These advantages likely help drive adoption by the industry.

### Low risk implementation

AI-driven automation can be implemented incrementally. “Small” hardware, such as cameras, tablets, or monitor screens, as well as the software components of the system, might be installed ahead of large hardware which may impact the facility’s floor plan. Facility staff can train and become familiar with the installed components. This approach lowers the risk of disruption to existing operations during installation and allows meat processors to “trial” the system. Once the reliability of the software components has been proven, more substantial equipment can be installed.

### Immediate value-add

Value for the meat processors can begin as soon as some components are installed. Value can be captured from the collection and analysis of data. The first components installed also likely begin to provide a small measure of assistance to workers. Workers whose workloads have been eased by the automated system can be reassigned to other areas of a production facility that are currently understaffed, or workers can move to value-added tasks not yet ready to be performed by AI.

### System improves over time

Because many AI-driven automation systems will incorporate machine learning components, these systems will learn from past performance and improve their accuracy over time. Software updates can also improve the performance or even expand the capabilities of an automated system without the downtime required for a new equipment installation.

### Ability to meet processor constraints

Meat processors point to several constraints that technology, including automated systems, must fit within for adoption. Available space, line speeds, downtime requirements, and the impact of the technology upstream and downstream are all concerns for processing managers. Artificial intelligence helps automation act within these constraints. In particular, software with the perception and decision-making abilities for multiple tasks can be attached to a single, dexterous robotic arm allowing it to perform more than one task. This increases the likelihood new systems can be built to fit within the constraints of existing processing operations.

### Reduced costs

Building one system that performs multiple tasks will also help lower the cost of AI-driven automation. In general, a single system performing multiple tasks will be expected to contain less hardware than a set of systems in which each performs a single task. Less equipment decreases the time and space required to install the system, lowering implementation costs. Fewer parts reduce maintenance time and replacement costs. AI-driven systems can also improve without incurring new equipment costs through updates to the software components. While the software needed for AI-driven automation will originally be costly to build, the long-range outlook suggests that such systems will ultimately be less expensive than alternative approaches.

## The Path to the Next Level of Optimization

### Gradual implementation

AI-driven automation systems will first augment human labor rather than immediately removing all workers from a task. The system will perform some physical labor or add some better decision-making capacity, thus allowing the worker to expand their production capacity or optimize their time and resource use. The AI-driven system will also learn from its human companions. In such a manner, Tyson’s computer vision system tracking poultry inventory augments the work done by human workers. Rather than tracking inventory manually, workers now confirm the accuracy of the system. The workers’ feedback has helped the system refine it’s algorithms to the point it is 20% more accurate than manual processes (Castellanos, 2020).

Gradually, AI-driven automation systems will take on more decision-making and transfer additional components of a task from the human companion to the automated system. It is likely the value of such a system will gradually increase as the system takes on more of the task for the worker or continues to increase its accuracy. Therefore, while AI-driven automation has value from its first installation, it likely reaches its full potential over time.

### Rethinking ROI

Calculating ROI as equal to reduced payroll costs often severely underestimates the actual return of an AI-driven automated system as it ignores productivity gains and other sources of return. As AI-driven automation is implemented into meat processing, a holistic view of the return on investment will need to be adopted to account for the full value of such systems.

### Asset utilization

Labor shortages sometimes lead to meat processing facilities running below capacity. Recently, facilities were reportedly operating six days a week to produce the same output as a five day schedule due to understaffing (Crews, 2021). AI-driven automation can help more fully utilize existing assets by decreasing the likelihood of such slowdowns. Through improved analytics to better predict maintenance needs or by operating without breaks for vacations or sick leave, it might also reduce facility downtime. Consequently, improved asset utilization likely contributes to the return on investment for AI-driven automation.

### Value capture

Labor availability sometimes impacts the product mix produced at meat processing facilities. With the introduction of AI-driven automation, workers from the automated area can be redeployed to tasks that help produce value-added products. The ability to capture additional value by producing products with optimal profitability contributes to the system’s return on investment.

### Increased sustainability

Well-designed and carefully implemented AI-driven automation can help stabilize meat processor operations, providing greater security for all stakeholders in the meat supply chain: from the producers, to the processors, to the consumers, and to government agencies seeking to secure the food supply. Such automation offers a chance to improve the sustainability of meat processing across all three pillars: social, economic, and environmental.

Improved health and safety and better working conditions for laborers in meat processing facilities can advance the social sustainability of meat processing. Jobs at meat processing facilities are not only unfilled because of an insufficient workforce availability, but also because of the often challenging nature of the work along with the less-than-desirable conditions (i.e., refrigerated environment) which makes the positions difficult to fill.

When jobs at meat processing facilities go unfilled, it can impact processing capacity and be destabilizing for the entire meat industry. Reduced processing capacity creates an increase in cattle supply, and puts downward pressure on fed cattle prices. Concurrently, decreased beef production, in conjunction with strong consumer demand, causes rising beef prices, widening the spread between cattle and beef prices (Aherin, 2021). Slowdowns at meat processing facilities during the COVID-19 pandemic had this effect. AI-driven automation can stabilize the industry for its many stakeholders and increase the economic sustainability of meat processing by reducing the number of slowdowns that result from unfilled and reluctantly filled positions at meat processing facilities.

AI-driven automation also has the potential to introduce additional efficiencies that further promote the stability and sustainability of meat processing operations. Increased consistency of output should reduce the amount of product needing to be reworked, reducing the associated cost and resource use. Decreased labor needs and increased stability of the facility’s workforce should also decrease training time and expenses as well as decrease overtime payments.

The environmental pillar of sustainability can be advanced by AI-driven automation through opportunities to decrease waste and add efficiencies that better use resources or reduce energy needs. Waste reduction may occur through a reduction in the need to rework products or improved package defect detection that decrease the amount of packaging material waste. The facility operator can also improve efficiency without needing to add employee and training cost, by providing task oriented robotic automation to areas that may be less critical, yet present an opportunity for adding value. An example of a value-added, energy saving sustainability task would be in hot fat carcass trimming.

Hot fat trimming removes fat from interior and exterior locations of the carcass shortly after slaughter before it enters the cooler. By removing fat destined for rendering while it is still warm, the facility avoids applying energy to chill the fat in the cooler, and then to reheat it for rendering. An AI-driven robot focusing on just the abdominal fat could remove anywhere from 1.5% to 4.0% of hot fat from a carcass (Holland, 2013); on a beef carcass weighing 400 kg, that would equate to 6–16 kg. of hot fat that can be removed prior to chilling. Implementing and consistently performing hot fat trimming reduces energy consumption of processing facilities and lowers energy costs.

### Changing mindsets, engaging with new stakeholders, and seizing opportunities

Facility managers can often find themselves in “firefighting mode,” dealing with only the most immediate and intense problems. Decisions to implement new equipment, then, often focus on solving a single pain point or bottleneck and putting out the biggest fire of the day. However, one AI-driven automation system has the potential to bring a new level of optimization and enhanced decision-making to multiple areas of a facility, from the fabrication floor all the way to the board room. As a result, time spent planning for system integration and developing high-level and long-range objectives for AI-driven automation yields a strong return. Meat processors will need to take on a new mindset and approach that separates the pursuit of AI-driven automation from traditional equipment purchase decisions.

Close alignment between the inventors of AI-driven automation and the end-users of the system starting early in the development process will be necessary to create systems that have a game-changing impact for the industry and fit into existing operations. Connections between major pain points, minor annoyances, and missed profit opportunities need to be mapped out and shared with the developers of AI-driven automation systems. Developers will need opportunities to collect data and metrics from meat processing facilities in order to both design and train AI-driven systems.

The processors who decide to engage early with the companies and startups building this technology will have the most say in how the technology is developed, what problems it prioritizes, and how well it integrates into existing operations. These meat processors will have recognized the opportunities AI-driven automation presents and the advantages of having the solutions that emerge tailored to their operations.

The difference between pursuing AI-driven automation and making traditional equipment purchases is not necessarily intuitive. More education for those in the meat processing industry about the opportunities and challenges presented by AI-driven automation is needed. In addition, some of the expertise to fully realize the potential of AI-driven automation for meat processing will likely need to be brought in from outside the meat industry.

### Building the right teams

Introducing AI-driven automation to meat processing depends on siloed knowledge being shared outside the usual channels. It will take traditional meat scientists and processing facility experts with a deep knowledge of meat processing working with engineers and developers who have expertise in the cutting-edge artificial intelligence and automation technologies used in other industries. These experts will need to come together with a curiosity to learn from one another and a willingness to share their particular expertise.

**Figure 3.**
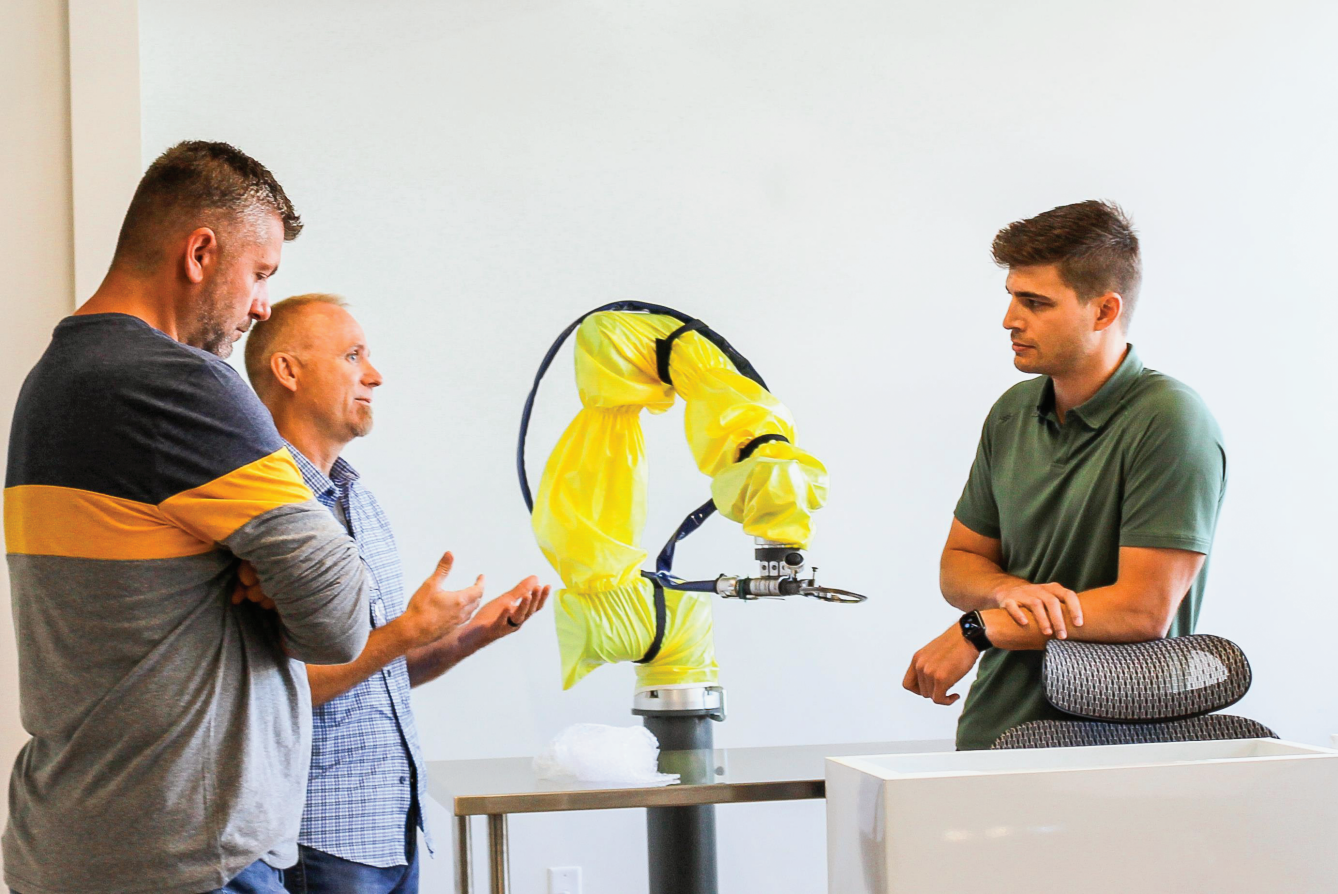
Meat scientists, engineers, software developers, and technology business leaders must collaborate on the development of AI-driven automation for meat processing.

However, engineering and scientific expertise is not enough. AI-driven automation is powered by software. As such, a talented and dedicated group of software developers is needed to integrate the knowledge of each industry and hardware expert into a durable, secure, high-performing software system ready for the rigors of meat processing facilities (Figure 3). The software architects will also need to bring their own expertise to the team, particularly in cybersecurity. As AI-driven automation is implemented, it is likely that pieces of equipment will need to communicate with one another and with cloud services. As a result, the security of the software against cyber attacks must be thoughtfully considered starting early in the development of AI-driven automation. Successful teams that will lead the development of next-level meat processing optimization and automation will bring all this engineering, software development, and meat science expertise together with a business team who understands the meat processor as a customer, and a skilled leadership team that knows how to build, scale, and maintain a technology company.

## Summary

The long-standing challenges to automation of meat processing, particularly those presented by the variability of meat products, are being solved by advancements in artificial intelligence technology, rather than advanced mechanical engineering. As a result, the next generation of automation will be a software-focused product rather than a hardware-focused product. Meat processors can achieve a new level of optimization with the help of this technology. However, the technology must be evaluated through a strategic lens that looks beyond reduced labor costs and accounts for the additional opportunities for return on investment and advancement of the meat industry.

*Conflict of interest statement.* The authors C. Barbar, R. Barbar, J. Bader, and B. Wondercheck are affiliated with Marble Technologies. The author P. Bass has provided meat science consultation for Marble Technologies.

## About the Authors

**Chafik Barbar** is the founder and CEO of Marble Technologies, founded to accelerate food system technologies for people and the planet. Chafik is a serial entrepreneur who has previously taken companies from idea to acquisition. He holds an MBA from MIT and has over 15 years of experience in software development for the Ag industry.

**Phillip D. Bass** is an assistant professor in the Department of Animal, Veterinary and Food Sciences at the University of Idaho. He earned his bachelor’s and master’s degree in animal science at California Polytechnic State University and his Ph.D. in meat science from Colorado State University. Phil spent several years with the Certified Angus Beef brand as their senior meat scientist prior to joining the faculty at the University of Idaho in 2017. Phil has an expertise in beef carcass fabrication and merchandising.

**Rachel Barbar** is the director of strategic initiatives at Marble Technologies, founded to accelerate food system technologies for people and the planet. Rachel’s work focuses on communicating complex scientific findings, and creating alignment across multiple stakeholders. Rachel earned a Master of Science in Genetic Counseling from the University of Wisconsin-Madison.

**Jordyn Bader** is the director of industry partnerships at Marble Technologies, founded to accelerate food system technologies for people and the planet. She earned her bachelor’s and master’s degree in agricultural economics at the University of Nebraska - Lincoln. Jordyn has spent her career in Ag Tech product development, and builds relationships with industry stakeholders to understand needs and opportunities within the Ag industry.

**Britany Wondercheck** is the director of business development at Marble Technologies, founded to accelerate food system technologies for people and the planet. Her career has been focused in Ag Tech product management, marketing, and customer account management. Britany earned her Bachelor of Science in Business Administration from the University of Nebraska at Omaha.

## References

[CIT0002] Aherin, D . 2021. Testimony on state of the beef supply chain: shocks, recovery, and rebuilding. U.S. House of Representatives Committee on Agriculture, Subcommittee on Livestock and Foreign Agriculture. [Accessed October 12, 2021]. https://docs.house.gov/meetings/AG/AG29/20210728/113973/HHRG-117-AG29-Wstate-AherinD-20210728.pdf.

[CIT0004] Barbut, S . 2014. Review: automation and meat quality-global challenges. Meat Sci. 96:335–345. doi:10.1016/j.meatsci.2013.07.002 23933632 10.1016/j.meatsci.2013.07.002

[CIT0005] Barbut, S . 2020. Meat industry 4.0: A distant future? Anim. Front. 10:38–47. doi:10.1093/af/vfaa038 33150010 10.1093/af/vfaa038 PMC7596804

[CIT0006] Boykin, C.A., L.C. Eastwood, M.K. Harris, D.S. Hale, C.R. Kerth, D.B. Griffin, A.N. Arnold, J.D. Hasty, K.E. Belk, D.R. Woerner, et al. 2017. National beef quality audit - 2016: in-plant survey of carcass characteristics related to quality, quantity, and value of fed steers and heifers. J. Anim. Sci. 95:2993–3002. doi:10.2527/tas2017.0029 28727109 10.2527/jas.2017.1543

[CIT0007] Byington, L . 2020. Meat processors expedite plans to implement robotics as pandemic increases pressure. FoodDive. [Accessed September 27, 2021]. https://www.fooddive.com/news/meat-processors-expedite-automation- as-pandemic-increases/588166/.

[CIT0008] Castellanos, S . 2020. Tyson takes computer vision to the chicken plant. Wall Street J. [Accessed September 9, 2021]. https://www.wsj.com/articles/tyson-takes-computer-vision-to-the-chicken-plant-11581330602?mod=djemlogistics_h.

[CIT0009] Choi, S., G. Zhang, T. Fuhlbrigge, T. Watson, and R. Tallian. 2013. Applications and requirements of industrial robots in meat processing. 2013 Institute of Electrical and Electronics Engineers International Conference on Automation Science and Engineering. doi:10.1109/CoASE.2013.6653967

[CIT0010] Crespi, J.M., and T.L. Saitone. 2019. Has specialization put a limit on how far cattle contracting can go?. Agriculture Policy Review, Winter 2019. Ames, Iowa: Center for Agricultural and Rural Development, Iowa State University. [Accessed September 22, 2021]. https://lib.dr.iastate.edu/cgi/viewcontent.cgi?article=1083&context=agpolicyreview.

[CIT0011] Crews, J . 2021. Tyson executives detail challenges facing chicken, pork operations in 2021. Meat+Poultry. [Accessed September 30, 2021]. https://www.meatpoultry.com/articles/24954-tyson-executives-detail-challenges-facing-chicken-pork-operations-in-2021.

[CIT0012] Emerson, M.R., D.R. Woerner, K.E. Belk, and J.D. Tatum. 2013. Effectiveness of USDA instrument-based marbling measurements for categorizing beef carcasses according to differences in longissimus muscle sensory attributes. J. Anim. Sci. 91:1024–1034. doi:10.2527/jas.2012-5514 23148250 10.2527/jas.2012-5514

[CIT0013] Frontmatec. 2021a. Automatic Loin Trimmer. [Accessed November 19, 2021]. https://www.frontmatec.com/media/6493/altl-1100-automatic-loin-trimmer-v3-3-gb_spread.pdf.

[CIT0014] Frontmatec. 2021b. Lamb shoulder machine. [Accessed November 19, 2021]. https://www.frontmatec.com/en/lamb-solutions/deboning-trimming/automatic-deboning-trimming/shoulder-machine.

[CIT0015] Gray, G., M. Moore, D. Hale, C. Kerth, D. Griffin, J. Savell, C. Raines, T. Lawrence, K. Belk, D. Woerner, et al. 2012. National beef quality audit - 2011: survey of instrument grading assessment of beef carcass characteristics. J. Anim. Sci. 90:5152–5158. doi:10.2527/jas.2012-5551 22952354 10.2527/jas.2012-5551

[CIT0016] Holland, R., and D. Loveday. 2013. Understanding yield grades and quality grades for value-added beef producers and marketers. Knoxville, Tennessee: University of Tennessee Institute of Agriculture Extension Bulletin SP 755.

[CIT0017] Howard, J . 2019. Artificial intelligence: implications for the future of work. Am. J. Ind. Med. 62:917–926. doi:10.1002/ajim.23037. 31436850 10.1002/ajim.23037

[CIT0018] Jackson, J.C., N. Castelo, and K. Gray. 2020. Could a rising robot force make humans less prejudice? Am. Psychol. 75:969–982. doi:10.1037/amp0000582 31916781 10.1037/amp0000582

[CIT0019] Kurzweil, R . 1990. The age of intelligent machines. Cambridge, MA: MIT Press.

[CIT0026] LeCun, Y., Y. Bengio, and G. Hinton. 2015. Deep learning. Nature. 521:436–444. doi:10/1038/nature14539. 10.1038/nature14539 26017442

[CIT0020] Moore, C.B., P.D. Bass, M.D. Green, P.L. Chapman, M.E. O’Connor, L.D. Yates, J.A. Scanga, J.D. Tatum, G.C. Smith, and K.E. Belk. 2010. Establishing an appropriate mode of comparison for measuring the performance of marbling score output from video image analysis beef carcass grading systems. J. Anim. Sci. 88:2464–2475. doi:10.2527/jas.2009-2593 20348376 10.2527/jas.2009-2593

[CIT0021] Salonen, A.O., and N. Haavisto. 2019. Towards autonomous transportation. Passengers’ experiences, perceptions and feelings in a driverless shuttle bus in Finland. Sustainability. 11:588–607. doi:10.3390/su11030588

[CIT0023] Tarakji, R . 2018. Are food manufacturers too slow in adopting new technology? Robot. Autom. News. [Accessed September 27, 2021]. https://roboticsandautomationnews.com/2018/03/27/are-food-manufacturers-too-slow-in-adopting-new-technology/16593/.

[CIT0024] Tedrake, R . 2021. Robotic manipulation: perception, planning, and control (course notes for MIT 6.881). https://manipulation.mit.edu/index.html [Downloaded, September 30, 2021].

[CIT0025] Zahariev, R.Z, and N. Valchkova. 2019. Existing robotics technologies for implementation of special education. In: M. Dimitrova, and H. Wagatsuma, editors. Cyber-physical systems. Hershey, PA: IMI Global;, p. 44–61. doi:10.4018/978-1-6684-3670-7.ch042

